# Can the 20 and 60 s All-Out Test Predict the 2000 m Indoor Rowing Performance in Athletes?

**DOI:** 10.3389/fphys.2022.828710

**Published:** 2022-06-03

**Authors:** Dario Cerasola, Daniele Zangla, Joseph N. Grima, Marianna Bellafiore, Angelo Cataldo, Marcello Traina, Laura Capranica, Nemanja Maksimovic, Patrik Drid, Antonino Bianco

**Affiliations:** ^1^ Department of Psychology, Educational Science and Human Movement, University of Palermo, Palermo, Italy; ^2^ Italian Rowing Federation, Turin, Italy; ^3^ Metamaterials Unit, Faculty of Science, University of Malta, Msida, Malta; ^4^ Department of Human Movement and Sport Sciences, University of Foro Italico, Rome, Italy; ^5^ Faculty of Sport and Physical Education, University of Novi Sad, Novi Sad, Serbia

**Keywords:** indoor rowing, youth rowers, anaerobic profile, all-out test, rowing race performance

## Abstract

**Purpose:** The purpose of this study was to look for a new, simple, and fast method of assessing and monitoring indoor race performance and to assess the relationship between 20 s, 60 s, and 2000 m indoor rowing performances of youth rowers to evaluate their anaerobic profile.

**Methods:** For three consecutive days, 17 young able-bodied male rowers (15.8 ± 2.0 years), performed three tests (20 s, 60 s, and 2000 m) on a rowing ergometer. Mean power (W_20_, W_60_, and W_2000_) and 2000 m time (t_2000_) were considered for the analysis. In addition, 14 athletes (15–18 years) performed a 20 s, 60 s, and 2000 m tests and used this as a control group. To define the anaerobic profile of the athletes, W_20_ and W_60_ were normalized as percentages of W_2000_. Associations between variables were determined by means of the Pearson correlation coefficient (*r*).

**Results*:*** Mean power decreased with increasing test duration (W_20_ = 525.1 ± 113.7 W; W_60_ = 476.1 ± 91.0 W; W_2000=_312.9 ± 56.0 W) and negative correlations emerged between t_2000_ (418.5 ± 23.1 s) and W_20_ (*r* = −0.952, *p* < 0.0001) and W_60_ (*r* = −0.930, *p* < 0.0001).

**Conclusion:** These findings indicate that W_20_ and W_60_ are significant predictors of 2000 m rowing ergometer performances. Furthermore, normalized W_20_ and W_60_ can be used to evaluate athletes and as a reference for planning anaerobic training sessions, on a rowing ergometer.

## Introduction

The sport of rowing, classically practiced on flat water, is a highly regulated sport in which athletes aim to cover the specified race distance as fast as possible, with the standard race distance being 2000 m in a straight-line distance as specified in the World Rowing Rule Book (FISA, 2020, http://worldrowing.com). In general, rowing performance, both on-water and indoor, depends on anthropometric (Podstawski et al., 2014), physiological (Secher, 1993; Ingham et al., 2003; Riechman et al., 2002; Bourdin et al., 2017), and psychological (Kellmann and Gunther, 2000; Shields et al., 2018) characteristics of the athletes, technical aspects (Den Hartigh et al., 2017), tactical strategies (Garland, 2005; Akça, 2014; Cerasola et al., 2018), and environmental conditions. Despite anthropometric and aerobic capacity being considered relevant for rowing performances (Ingham et al., 2003; Cosgrove et al., 1999; Smith and Hopkins, 2012), the anaerobic metabolism is crucial to allow athletes to accomplish fast starts and final spurts, which could vary in terms of duration ranging from 20 to 60 s in relation to the race strategy (Garland, 2005; Maestu et al., 2006; Cataldo et al., 2015; Martin and Tomescu, 2017). In fact, recent evidence shows that power output, and not just aerobic capacity, could be an important predictor of race outcomes (Izquierdo-Gabarren et al., 2010; Lawton et al., 2011; Cataldo et al., 2015; Bourdin et al., 2017; Martin and Tomescu, 2017; Cerasola et al., 2020).

In agreement with the reported contribution of the aerobic and anaerobic metabolisms to rowing races (Secher, 1993), the training plan of successful rowers normally encompasses 65–70% aerobic exercises and 30–35% anaerobic ones (Maestu et al., 2006). To evaluate athletes and monitor their training plan, coaches routinely assess the boat speed with specific devices such as Global Position System (GPS). Also closely monitored is the performance of athletes on the rowing ergometer, typically through the rowing ergometer speed (measured with an integrated computer) or other parameters which can be accurately reported as a function of time or distance rowed. In fact, from all the possible parameters, most coaches prefer to consider “speed” as the most crucial parameter because specific training sessions are planned in considering different percentages of race speed (Jensen, 1994). Furthermore, coaches often make use of standardized indoor rowing tests to monitor the effects of their training plans.

Measuring changes in performance is important not only for monitoring the progress of rowers during training, but also to further develop the knowledge of the sport through research assessing the effect of training and other interventions. For example, there seems to be a consensus amongst the researchers and practitioners in the field that anaerobic tests for measuring mean and peak power outputs on a rowing ergometer show a high positive correlation with respect to “all-out” 2000 m indoor rowing performance in elite athletes (Riechman et al., 2002; Cataldo et al., 2015; Bourdin et al., 2017). In contrast, the assessment of anaerobic power in youth athletes is still somewhat controversial (Mikulic, 2008; Mikulic et al., 2009; Cataldo et al., 2015; Maciejewski et al., 2016) probably due to large variability in body dimension and in technical proficiency of this population (Mikulic, 2008; Maciejewski et al., 2016). At this crucial age, in a number of countries, coaches, and sports associations are encouraged to design their training schedule on Balyi’s “Long Term Athletic Development” (LTAD) model which suggests that the athletic potential of youngsters should be carefully aligned with their biological growth. The model suggests that there should be a focus to optimize performance “longitudinally” and recognize the importance of very particular and specific developmental “windows of opportunity” time periods. (Ford et al., 2011). At the same time, youth athletic success is considered relevant for talent detection, selection, and development (Capranica and Millard-Stafford, 2011). In rowing, youth competitions are organized at local, regional, national, continental, and world levels, and are also included in the Youth Olympic Games.

Experts agree that, given the specific needs of this special population, the training and competitions which are standard for senior and elite athletes may need to be adapted. To meet the characteristics of youth athletes and to facilitate the development of their technical and tactical skills, youth competitions could, ideally, encompass also 1,000 m and 1,500 m distances (Maciejewski et al., 2016). In particular, the literature highlighted that in rowing races effort regulation depends on the performance level, with marked end-spurts occurring more often at sub-elite levels (Brown et al., 2010). In considering the developmental phase of youth rowers, several authors considered relevant the evaluation of the anaerobic capability of youth athletes (Mikulic, 2008; Mikulic et al., 2009; Mikulic and Markovic 2011; Cataldo et al., 2015; Maciejewski et al., 2016; Cerasola et al., 2020).

The aim of this study was to look for a new method, simple and fast, of assessing and monitoring indoor race performance and to investigate the relationship between the fixed-time 20 s and 60 s all-out tests and the fixed-distance 2000 m indoor rowing performance in youth athletes. In particular, this work will test the hypothesis that mean power performance during 20 s and 60 s all-out tests could predict the 2000 m performance of youth rowers.

## Methods

### Participants

The institutional review board of the University of Palermo approved the within-subjects experimental design, which included 17 male youth (age: 15.8 ± 2.0 years; height 176.1 ± 7.8 cm; body mass: 70.9 ± 10.0 kg) rowers affiliated to the Italian Rowing Federation and finalists in the Italian Men’s Junior Rowing Championship (15–18 years). Furthermore, an independent sample of 14 youth rowers (15–18 years) was considered to cross-validate the findings of the experimental sample. After a detailed explanation of the nature and purpose of the study, written informed consent to participation was obtained from the athletes and their parents before the commencement of the study. Athletes had at least three 3) years of previous rowing training consisting of 18–20 h week^−1^ according to the recommendations of the Italian Rowing Federation FIC. In addition, 13 older male high-level athletes (21.1 ± 0.8 years) performed similar 20 s, 60 s, and 2000 m tests, the results of which were used to preliminarily assess whether the tests can be applied to athletes of a different age group.

### Experimental Design

During the pre-competitive period of the 2019–2020 season, the experimental period included three sessions, with a “rest day” in-between, during which the participants were required to perform a 20 s, a 60 s, and 2000 m all-out tests, respectively. Participants were habituated to these tests, routinely administrated during the season by their coach. Prior to the test session, body mass and stature were measured by means of a stadiometer and an electronic scale (SECA, Germany). This was followed by a 15-min standard warm-up eliciting ∼140 beats·min^−1^. Throughout the test, the rowers received verbal encouragement from their coach to perform their best.

The tests were performed on a Concept2 rowing ergometer (mod. D, Concept2, Morrisville, United States, fitted with a PM5 monitor) with a 120-drag factor. The apparatus provided information, amongst other things, on the mean power for the 20 s, 60 s, and 2000 m events, W_20_, W_60_, and W_2000_, measured in watts (W), where 1 W = 1 J s^−1^ and the 2000 m performance time (t_2000_, s). Absolute W_20_ and W_60_ values were normalized relative to body weight. Finally, the mean speed (V_2000_, m^.^s^−1^) of the 2000 m performance was also calculated. The same sequence was used in the counter-test group.

### Statistical Analysis

Statistical significance was accepted with an alpha level of *p* ≤ 0.05. Data are presented as “means ± SD”. At first, the Kolmogorov-Smirnov and Shapiro–Wilk normality tests were used to assess the normal distribution of the experimental variables W_20_, W_60_, W_2000_, t_2000_, and V_2000_. Pearson correlation coefficients (r) and linear regression analysis (*R* ^2^) were used to determine the association between t_2000_, W_60_, and W_20_ variables. According to the literature Hopkins (2000), *R* ^2^ was considered trivial, small, moderate, large, very large, nearly perfect, and perfect for values <0.01, >0.01–0.09, >0.09–0.25, >0.25–0.49, >0.49–0.81, >0.81, 1.0, respectively. Furthermore, a stepwise regression analysis was used to examine the relationship between V_2000_, absolute W_20_, and W_60_. Finally, to plot the anaerobic profile of the youth rowers, absolute W_20_ and W_60_ values were expressed as percentages of W_2000_.

## Results

Absolute and relative W_20_ values were 525.1 ± 113.7 W (range: 340–690 W) and 7.4 ± 0.9 W kg^−1^ (range: 6.17–8.46 W kg^−1^), respectively; whilst absolute and relative W_60_ values of 476.1 ± 91.0 W (range: 333–649 W) and 6.7 ± 0.8 W kg^−1^ (range: 6.22–7.44 W kg^−1^), respectively. The 2000m indoor rowing performance lasted 418.5 ± 23.1 s, with a V_2000_ of 4.8 ± 0.3 m s^−1^ and absolute W_2000_ of 312.9 ± 56.0 W. These values substantiate their good athletic level in relation to age. Anaerobic parameters always showed negative relationships (*p* < 0.001) with respect to t_2000_, resulting in higher absolute values with respect to relative values (Table 1).

**TABLE 1:** Correlation between 2000 m rowing ergometer performance time (t_2000_) and anaerobic characteristics of the 17 participants.

	*t*_2000_ vs. absolute mean power		*t*_2000_ vs. relative mean power	
	W_20_ (W)	W_60_ (W)	W_20_ (W kg^−1^)	W_60_ (W kg^−1^)
*R*	−0.920	−0.914	−0.746	−0.615
*R2*	−0.847	0.836	0.836	0.372
*P*	< 0.0001	< 0.0001	0.0006	0.0008

**FIGURE 1:**
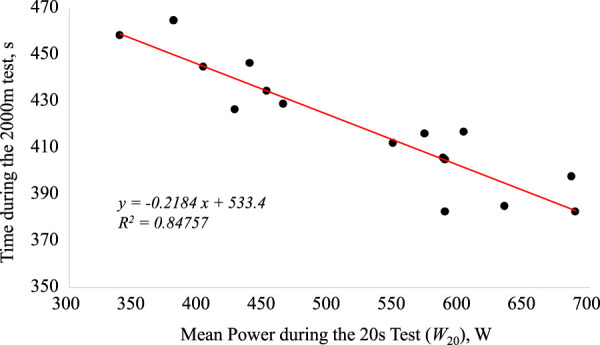
The relationship between the 2000 m performance time (*t*_2000_) and mean power (*W*_20_) during the 20 s all-out test.

**FIGURE 2:**
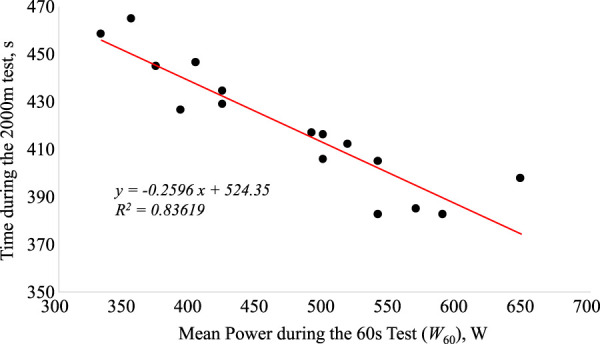
The relationship between the 2000 m performance time (*t*_2000_) and mean power (*W*_60_) during the 60 s all-out test.

**FIGURE 3:**
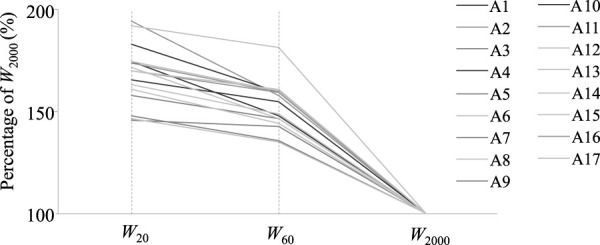
Anaerobic power profiles of the 17 youth athletes expressed through the percentages of the mean power *W*_20_ and *W*_60_ during the 20 and 60s tests with respect to the 2000m actual performance.

Figure 1 and Figure 2 show the regression equations between t_2000_ and absolute W_20_ and W_60_ values, respectively. Significant correlations were observed between V_2000_ and W_20_ (r = 0.95; *p* < 0.004) and W_60_ (r = 0.98; *p* < 0.0005). The stepwise multiple regression identified the prediction equation V_2000_ = 2.795 - (0.0005303 *W_20_) + (0.004680 *W_60_), with W_20_, and W_60_ accounting for 96.8% of the variance of V_2000_ (*p* < 0.01). The cross-validation of the prediction equation with the independent sample of youth rowers showed a high correlation between actual and predicted rowing speed (r = 0.98; *p* = 0.0005) and prediction limits ranging from -0.12 to 0.11 m s^−1^ (-2.74–2.78%). Figure 3 shows the anaerobic profile of the youth rowers, with W_20_ and W_60_, presented as percentages of W_2000_. In particular, W_20_ showed the highest values (168.1 ± 14.1%) with respect to W_60_ (153.1 ± 11.3%). Also reported in Table 2, as discussed below, are the findings from preliminary tests on a cohort of older athletes.

**TABLE 2:** The measured *W*_20_ and *W*_60_ values of the 13 additional older volunteers (mean age 21.1 ± 0.8) and the percentage difference between the achieved 2000m event speed and model predicted speed for a 2000m event.

*W*_20_ (W)	*W*_60_ (W)	Achieved speed in 2000 m event (ms^−1^)	Predicted speed in 2000 m event (ms^−1^)	Speed difference (%)
847.2 ± 45.9	710.6 ± 34.4	5.49 ±0.1	5.67 ± 0.1	0.97 ± 0.02

## Discussion

The main findings of this study were the definition of 1) the mathematical model to predict actual 2000 m indoor rowing performance and 2) the anaerobic profile of youth rowers. Furthermore, this study substantiates the significant correlation between average power outputs of 20 and 60 s (Cataldo et al., 2015; Cerasola et al., 2020), and the time to complete 2000 m indoor rowing performance.

In the present study, t_2000_ resulted coherent with the literature of Cerasola et al. (2020); Cataldo et al. (2015); Mikulic et al. (2009). This is an important finding, especially considering the athletic level of the participants but needs to be considered in the context of the present knowledge in this field. For example, despite the well-known and relevant aerobic contribution to rowing (Cosgrove et al., 1999), estimated at ∼30–35% (Secher, 1993), it is now also known that the VO_2max_ parameter presents limited predictive capability with some authors (Maestu et al., 2006) proposing anaerobic power as an overall index of 2000 m performance. As an alternative, Cerasola and co-workers have proposed that coaches should consider W_20_ and W_60_ relevant parameters for monitoring the capability of their athletes to perform high-intensity phases, which are crucial during the first part of the race for increasing the boat speed to gain advantages over the opponents and to sustain a final rush to achieve the best ranking when the boats are “tip to tip”, respectively (Garland, 2005; Cerasola et al., 2018). In fact, data was collected during a 30 s modified Wingate test (Mikulic et al., 2009), a 20 s all-out rowing support mean power (Cataldo et al., 2015) and a 60 s all-out rowing support mean power (Cerasola et al., 2020) proved to be as a better predictor of the time to complete 2000 m with respect to VO_2max_. Furthermore, Egan-Shuttler et al*.* (2014; 2017) show that the force is relevant to increasing the rowing performance during the 500 m time trial.

This present study adds more confidence to this claim favoring the use of such 20 and 60 s all-out power tests. In fact, by investigating 20 and 60 s all-out anaerobic performances of youth rowers, the present study confirms and implements these results and indicates that this newly developed model is safe (i.e., youth rowers were able to perform the test without any personal risk), accurate and effective (i.e., the 20 and 60 s are very simple tests, therefore all rowers can express the maximum performance) in predicting 2000 m rowing ergometer performance. This finding becomes even more relevant in view of the fact that, in general, indoor rowing performances are considered good predictors of on-water 2000 m time of elite youth rowers, with a standard error of the estimate ranging between 2.6 and 7.2% (Smith and Hopkins, 2012).

More specifically, W_20_ and W_60_ resulted in the most significant variables for predicting t_2000_, probably due to the relevant combined contribution of the alactacid and lactacid anaerobic metabolisms, respectively (Jensen, 1994). According to Steinacker (1993), senior elite athletes compete in a typical rowing race in the single scull reach a mean power of 450–600 W during the first 10 s of the initial phase and 400–500 W during the 60 s of the final phase, respectively. In the present study, youth athletes reached similar mean power values during the 20 s (e.g., 340–690 W) and 60 s tests (e.g., 333–649 W), which substantiate their good athletic level.

Finally, with respect to the literature (Cosgrove et al., 1999; Riechman et al., 2002; Cataldo et al., 2015; Cerasola et al., 2020) the integration of W_20_ and W_60_ in the mathematical model for the prediction of 2000 m speed provides a better evaluation of performance (e.g., < 3% error) and further evidence on the relevance of testing anaerobic capacity of youth athletes.

Before concluding it is important to highlight the strengths and limitations of the present work.

The main strengths of this work are the novelty and implications of the findings which are of significant practical importance within the context of rowing federations and clubs as monitoring tests and talent identification.

To sustain the 5.5–7.0 min of rowing competitions both aerobic and anaerobic training are important components of training programs, with training stimuli differing depending on the type, length, and intensity of each session (Maestu et al., 2006). In particular, during the preparation period, the main goal of rowing training is to build up the aerobic endurance of athletes, whereas during the competition period the focus is on the development of the aerobic and anaerobic components. In general, training intensities can be defined as percentages of power at race pace, considering intensities of 110–180% to develop anaerobic capacity, 90–105% aerobic transportation, 75–85% anaerobic threshold, and 65–70% aerobic utilization (Jensen, 1994). Despite the evaluation of the aerobic capacity of rowers is well documented in the literature and commonly used to structure individualized training, information on the anaerobic capacity is much less evaluated (Izquierdo-Gabarren et al., 2010). To establish appropriate training stimuli in relation to the developmental phase and athletic level of the youth rowers, coaches are urged to routinely monitor their progress to structure sound training sessions (Capranica and Millard-Stafford, 2011). Considering that youth rowers habitually undergo a training volume of 18–20 h week^−1^ (FIC, 2020) (http://www.canottaggio.org) the evaluation of their anaerobic capacity could be effectively achieved by means of computerized rowing ergometers that provide information on time, power, speed, and stroke rate. Thus, the anaerobic power curve shown in this study could help the coaches to define different levels for anaerobic intensities, to monitor the effectiveness of their training plan, and to identify and promote talents by predicting athletic success in 2000 m competitions (Maestu et al., 2006). In considering that this study is limited to the peculiar sample of finalists in the Italian Men’s Junior Rowing Championship, further studies are needed to verify whether these tests are useful for the evaluation of youth rowers of different athletic levels, ages, and sex.

Talent identification and promotion of youth athletes is a complex phenomenon, often based on the assessment of their sport-specific performance. Despite rowing also several environmental (e.g., wind, water currents, and temperature) and tactical aspects of successful performances should be considered, comparisons between the time needed to cover distances during on-water and indoor races presented limited discrepancies (Izquierdo-Gabarren et al., 2010; Vogler et al., 2010). Therefore, 2000 m time indoor trials have become an important selection tool for national rowing organizations (Smith and Hopkins, 2012), with coaches ranking athletes on the team in a controlled environment. The findings of this study not only provided valuable insights into the evaluation of the anaerobic capability of youth athletes but also a good prediction model for their 2000 m speed performances. Thus, coaches should consider the 20 and 60 s all-out tests also for talent identification and progress.

### Strengths and Limitations

Like any other study, this work has its own limitations. In particular, the results for the mean power values W_20_ and W_60_ had associated with them a large standard deviation since the youth athletes reached quite a wide range of mean power values during the 20 s (340–690 W) and 60 s tests (333–649 W). This profile of data, unfortunately, is unavoidable when testing athletes of age (age 15.8 ± 2.0 years). At such age, different athletes are expected to be at different levels of their biological development with the resultant variations in somatic growth, body composition, and somatic proportions (height 176.1 ± 7.8 cm; body mass: 70.9 ± 10.0 kg) that could have a direct influence on aerobic and anaerobic sports performances (Malina et al., 2004; Beunen and Malina, 2008). It must furthermore, it should be highlighted that the effectiveness of such a method applies within the realm of this study and to the characteristic of the participants tested. Indeed, as the participants tested were young athletes, all males (age range: 15.8 ± 2.0 years old), any speculation on the possibility to apply such a method to a different gender, age and athletes level groups require further testing. In this respect, it is encouraging that preliminary tests we conducted on the second group of 13 older volunteer high-level adult athletes (male, age: 21.1 ± 0.8; height 191.2 ± 6.6 cm; body mass: 88.3 ± 4.9 kg) who underwent tests to preliminary assess the transferability of the tests to different age categories suggested that these predictive tools are still applicable. In these additional tests, we calculated the percentage difference between the achieved speed in the 2000 m event and the predicted speed computed using the 20 and 60 s all-out tests according to previous stepwise multiple regression and they found that the achieved speed measured in 2000 m all-out event, 5.49 ± 0.1 m s^−1^, only differed by 0.97% compared to the speed estimated from the predictive tests, 5.67 ± 0.1 m s^−1^ (see Table 2). In view of these positive findings, we hope this work will provide an impetus to other researchers to collect additional data from different cohorts so as to further rigorously validate the reliability of the 20 and 60 s tests as a monitoring tool for the standard 2000 m event. Such further validation studies could also look into aspects such as the correlation between trials, measures of reliability coefficient, Cronbach’s alpha, coefficient of variation between trials on a particular day and/or between days, etc. Given this is a newly proposed paradigm, such validation studies are important before these tests can be implemented universally worldwide as a training monitoring tool. Furthermore, the present study was focused on predicting the ‘mean power’, but it is well known that other parameters, such as the “peak power” (i.e., highest value observed) are also useful training monitoring tools (Egan-Shuttler et al., 2014; 2017). In fact, peak and mean are, sometimes, considered to be complementary approaches to measure rowing performance and the rowing community has not yet determined which of these might be more valuable, particularly in view of the fact athletes often take part in different types of events. It is thus essential that future studies would also look at the peak power, as well as other variables which could be predictable through the quick 20 and 60 s tests.

Nevertheless, it is envisaged that such limitations can be addressed through further studies and tests, ideally conducted by independent researchers, particularly in view of the fact that a major strength of this work is the relative simplicity to carry out the proposed 20 and 60 s tests, their role in the talent identification process and as a monitoring tool, and their ability to make accurate and fast predictions. Moreover, the proposed tests, particularly the 20 s test, are quick and easy to do, without fatiguing the athletes, or creating anxiety hence allowing more time/rigorous training to follow within the same session and, at the same time, allowing tracking of athletes. This makes them ideal for routine monitoring of athletes: a 20 s all-out row will practically go unnoticed and is hardly going to make an impact on the rest of the training session.

## Conclusion

By not requiring expensive equipment, specific scientific expertise, and long duration, the 20- and 60-s tests could be a valuable tool to routinely assess youth athletes during training sessions. Furthermore, they could be considered a more feasible and accurate option to predict 2000 m performances with respect to the assessment VO_2max_, which requires expensive equipment, invasive methods, and some attendant risks might be present. Therefore, the proposed mathematical model could offer coaches a simple workout to propose to athletes to predict their performance, with a frequency greater than the test of 2000 m that is not commonly used in training due to the great effort required.

## Data Availability

The raw data supporting the conclusion of this article will be made available by the authors, without undue reservation.

## References

[B1] Akça F. (2014). Prediction of Rowing Ergometer Performance from Functional Anaerobic Power, Strength and Anthropometric Components. J. Hum. Kinet. 41, 133–142. 10.2478/hukin-2014-0041 25114740 PMC4120446

[B2] Beunen G. Malina R. M. (2008). Growth and Biologic Maturation: Relevance to Athletic Performance. The Young Athlete 13, 3–17. 10.1002/9780470696255.ch1

[B3] Bourdin M. Lacour J.-R. Imbert C. Messonnier L. (2017). Factors of Rowing Ergometer Performance in High-Level Female Rowers. Int. J. Sports Med. 38, 1023–1028. 10.1055/s-0043-118849 28965342

[B4] Brown M. R. Delau S. Desgorces F. D. (2010). Effort Regulation in Rowing Races Depends on Performance Level and Exercise Mode. J. Sci. Med. Sport 13, 613–617. 10.1016/j.jsams.2010.01.002 20227342

[B5] Capranica L. Millard-Stafford M. L. (2011). Youth Sport Specialization: How to Manage Competition and Training? Int. J. Sports Physiol. 6, 572–579. 10.1123/ijspp.6.4.572 22174125

[B6] Cataldo A. Cerasola D. Russo G. Zangla D. Traina M. (2015). Mean Power during 20 Sec All-Out Test to Predict 2000 M Rowing Ergometer Performance in National Level Young Rowers. J. Sports Med. Phys. Fitness. 55, 872 24921619

[B7] Cerasola D. Bellafiore M. Cataldo A. Zangla D. Bianco A. Proia P. (2020). Predicting the 2000‐m Rowing Ergometer Performance from Anthropometric, Maximal Oxygen Uptake and 60‐s Mean Power Variables in National Level Young Rowers. J. Hum. Kinet. 75, 77–83. 10.2478/hukin-2020-0038 33312296 PMC7706680

[B8] Cerasola D. Cataldo A. Bellafiore M. Traina M. Palma A. Bianco A. (2018). Race Profiles of Rowers during the 2014 Youth Olympic Games. J. Strength Cond. Res. 32, 2055–2060. 10.1519/JSC.0000000000002364 29939950

[B9] Cosgrove M. J. Wilson J. Watt D. Grant S. F. (1999). The Relationship between Selected Physiological Variables of Rowers and Rowing Performance as Determined by a 2000 M Ergometer Test. J. Sports Sci. 17, 845–852. 10.1080/026404199365407 10585164

[B10] Den Hartigh R. J. Marmelat V. Cox R. F. (2017). Multiscale Coordination between Athletes: Complexity Matching in Ergometer Rowing. Hum. Mov. Sci. 57, 434–441. 10.1016/j.humov.2017.10.006 29107321

[B11] Egan-Shuttler J. D. Edmonds R. Eddy C. O'Neill V. Ives S. J. (2014). Beyond Peak, a Simple Approach to Assess Rowing Power and the Impact of Training: A Technical Report. Int. J. Exerc. Sci. 12 (6), 233 10.70252/YNWB9446 PMC6355124 30761208

[B12] Egan-Shuttler J. D. Edmonds R. Eddy C. O’Neill V. Ives S. J. (2017). The Effect of Concurrent Plyometric Training versus Submaximal Aerobic Cycling on Rowing Economy, Peak Power, and Performance in Male High School Rowers. Sports Med. - Open 3 (1), 7. 10.1186/s40798-017-0075-2 28150178 PMC5288420

[B13] FIC (2020). Italian Rowing Federation. Available at: http://www.canottaggio.org (Accessed March 15, 2020).

[B14] FISA (2020). World Rowing. Available at: http://www.worldrowing.com/coachs/ (Accessed March 15, 2020).

[B15] Ford P. De Ste Croix M. Lloyd R. Meyers R. Moosavi M. Oliver J. (2011). The Long-Term Athlete Development Model: Physiological Evidence and Application. J. Sports Sci. 29 (4), 389–402. 10.1080/02640414.2010.536849 21259156

[B16] Garland S. W. (2005). An Analysis of the Pacing Strategy Adopted by Elite Competitors in 2000 M Rowing. Br. J. Sports Med. 39, 39–42. 10.1136/bjsm.2003.010801 15618339 PMC1725010

[B17] Hopkins W. G. (2000). Measures of Reliability in Sports Medicine and Science. Sports Med. 30, 1–15. 10.2165/00007256-200030010-00001 10907753

[B18] Ingham. S. Whyte. G. Jones. K. Nevill. A. (2002). Determinants of 2,000 M Rowing Ergometer Performance in Elite Rowers. Eur. J. Appl. Physiol. 88, 243–246. 10.1007/s00421-002-0699-9 12458367

[B19] Izquierdo-Gabarren M. de Txabarri Expósito R. G. de Villarreal E. S. S. Izquierdo M. (2010). Physiological Factors to Predict on Traditional Rowing Performance. Eur. J. Appl. Physiol. 108, 83–92. 10.1007/s00421-009-1186-3 19756709

[B20] Jensen K. (1994). Test Procedures for Rowing. FISA Coach 5, 1–6.

[B21] Kellmann M. Gunther K.-D. (2000). Changes in Stress and Recovery in Elite Rowers during Preparation for the Olympic Games. Med. Sci. Sports Exerc. 32, 676–683. 10.1097/00005768-200003000-00019 10731012

[B22] Lawton T. W. Cronin J. B. McGuigan M. R. (2011). Strength Testing and Training of Rowers. Sports Med. 41, 413–432. 10.2165/11588540-000000000-00000 21510717

[B23] Maciejewski H. Rahmani A. Chorin F. Lardy J. Giroux C. Ratel S. (2016). The 1,500-m Rowing Performance Is Highly Dependent on Modified Wingate Anaerobic Test Performance in National-Level Adolescent Rowers. Pediatr. Exerc. Sci. 28, 572–579. 10.1123/pes.2015-0283 27633491

[B24] Mäestu J. Jürimäe J. Jürimäe T. (2005). Monitoring of Performance and Training in Rowing. Sports Med. 35, 597–617. 10.2165/00007256-200535070-00005 16026173

[B25] Malina R. Bouchard C. Bar-Or O. (2004). “Growth, Maturation, and Physical Activity.” in Human Kinetics. 2nd Ed. USA (Champaign, IL).

[B26] Martin S. A. Tomescu V. (2017). Energy Systems Efficiency Influences the Results of 2,000 M Race Simulation Among Elite Rowers. Clujul Med. 90, 60–65. 10.15386/cjmed-675 28246499 PMC5305090

[B27] Mikulic P. (2008). Antropometric and Physiological Profiles of Rowers of Varying Ages and Ranks. Kinesiology 40, 80–88.

[B28] Mikulic P. Markovic G. (2011). Age- and Gender-Associated Variation in Maximal-Intensity Exercise Performance in Adolescent Rowers. Int. J. Sports Med. 32, 373–378. 10.1055/s-0031-1271762 21380965

[B29] Mikulić P. Ružić L. Marković G. (2009). Evaluation of Specific Anaerobic Power in 12-14-Year-Old Male Rowers. J. Sci. Med. Sport 12, 662–666. 10.1016/j.jsams.2008.05.008 18762452

[B30] Podstawski R. Choszcz D. Konopka S. Klimczak J. Starczewski M. (2014). Anthropometric Determinants of Rowing Ergometer Performance in Physically Inactive Collegiate Females. Biol. Sport 31, 315–321. 10.5604/20831862.1133936 25609890 PMC4296840

[B31] Riechman S. Zoeller R. Balasekaran G. Goss F. Robertson R. (2001). Prediction of 2000 M Indoor Rowing Performance Using a 30 S Sprint and Maximal Oxygen Uptake. Rjsp 20, 681–687. 10.1080/026404102320219383 12200919

[B32] Secher N. H. (1993). Physiological and Biomechanical Aspects of Rowing. Sports Med. 15, 24–42. 10.2165/00007256-199315010-00004 8426942

[B33] Shields M. R. Brooks M. A. Koltyn K. F. Kim J.-S. Cook D. B. (2017). Cognitive Resilience and Psychological Responses across a Collegiate Rowing Season. Med. Sci. Sports Exerc. 49, 2276–2285. 10.1249/MSS.0000000000001363 28682806

[B34] Smith T. B. Hopkins W. G. (2012). Measures of Rowing Performance. Sports Med. 42, 343–358. 10.2165/11597230-000000000-00000 22401296

[B35] Steinacker J. M. (1993). Physiological Aspects of Training of Rowing. Int. J. Sports Med. 14, 3–10. 10.1055/s-2007-1021214 8262704

[B36] Vogler A. J. Rice A. J. Gore C. J. (2010). Physiological Responses to Ergometer and On-Water Incremental Rowing Tests. Int. J. Sports Physiol. Perform. 5, 342–358. 10.1123/ijspp.5.3.342 20861524

